# Elementary Reactions and Their Role in Gas-Phase Prebiotic Chemistry

**DOI:** 10.3390/ijms10052304

**Published:** 2009-05-19

**Authors:** Nadia Balucani

**Affiliations:** Dipartimento di Chimica, Università degli Studi di Perugia, 06123 Perugia, Italy; E-Mail: nadia.balucani@unipg.it; Tel. +39-075-585-5513; Fax: +39-075-585-5606

**Keywords:** gas-phase prebiotic chemistry, nitriles, organosulfur compounds

## Abstract

The formation of complex organic molecules in a reactor filled with gaseous mixtures possibly reproducing the primitive terrestrial atmosphere and ocean demonstrated more than 50 years ago that inorganic synthesis of prebiotic molecules is possible, provided that some form of energy is provided to the system. After that groundbreaking experiment, gas-phase prebiotic molecules have been observed in a wide variety of extraterrestrial objects (including interstellar clouds, comets and planetary atmospheres) where the physical conditions vary widely. A thorough characterization of the chemical evolution of those objects relies on a multi-disciplinary approach: 1) observations allow us to identify the molecules and their number densities as they are nowadays; 2) the chemistry which lies behind their formation starting from atoms and simple molecules is accounted for by complex reaction networks; 3) for a realistic modeling of such networks, a number of experimental parameters are needed and, therefore, the relevant molecular processes should be fully characterized in laboratory experiments. A survey of the available literature reveals, however, that much information is still lacking if it is true that only a small percentage of the elementary reactions considered in the models have been characterized in laboratory experiments. New experimental approaches to characterize the relevant elementary reactions in laboratory are presented and the implications of the results are discussed.

## 1. Introduction

In the sequence of steps which are believed to have led from elementary particles to the emergence of life, an important one is certainly the formation of simple prebiotic molecules from parent species abundant in the Universe. The aggregation of H, O, C, N, S and other atoms into molecules and the subsequent chemical evolution are occurring also now in the Universe, as witnessed by the identification of more than one hundred molecules in the interstellar clouds (encompassing also prebiotic molecules such as hydrogen cyanide, glycolaldehyde, formamide and even, tentatively, glycine) and by the gas-phase chemical evolution of the atmospheres of several solar objects such as Titan. Certainly, these processes might seem relatively simple compared to the other unknown phenomena that have led to the first living organisms. Nevertheless, the formation mechanisms of many of the observed gaseous prebiotic molecules and radicals are far from being understood, while a comprehension of those processes can certainly help to set the stage for the emergence of life to occur.

If we compare the elemental composition of the Universe and that of a living cell, a remarkable difference is evident, especially as far as the abundances of carbon and nitrogen are concerned. And not only is the elemental composition different, but also the type of molecules: mostly large and complex in a cell, rare and simple in the Universe. Clearly a chemical differentiation has taken place, firstly in the formation and evolution of solar systems in star-forming regions and then in the passage from inanimated matter to living entities. A series of intermediate molecular species, characterized by some complexity and all the appropriate “ingredients”, might be the link between these extremely different environments. In Table 1 are grouped a series of molecules which can be synthesized in abiotic processes (*e.g.* in the gas phase), but might be prebiotic in nature [1,2] and which have been identified in extraterrestrial gaseous environments (of course this does not mean that they are permanent gases under the typical conditions of Earth). For instance, gaseous molecules containing a C-N bond (such as HCN, HC_3_N and CH_3_CN) are potential precursors of aminoacids or nucleobases in the presence of water, while molecules containing a C-O bond might be the building blocks for the synthesis of sugars and aminoacids (notable examples are glycol-aldehyde and ethylene glycol).

Finally, gaseous molecules with multiple C-C bonds provide the skeleton for long carbon chain molecules and are possible precursors of polycyclic aromatic hydrocarbons (PAHs). Other molecules which might be synthesized in the gas phase are water, ammonia, formamide, sulfur and phosphorus compounds *etc.*

How these molecules, or even more complex ones, could have reached or been developed on our planet is a matter of debate. Essentially two possible scenarios have been suggested so far, the *endogenous* synthesis and the *exogenous* synthesis [3,4]. According to some theories dating back to the ‘warm little pond’ suggested by Darwin and the ‘primordial soup’ idea by Oparin, the synthesis of organic molecules occurred directly in our planet starting from simple parent molecules (such as N_2_ or NH_3_, H_2_O or H_2_, CH_4_ or CO_2_ and others) [5–9] which constituted a sort of secondary atmosphere of Earth, after the primordial one was swept away during the first period of the solar system life. There are some discussions on the composition of the atmosphere back then, which could be in a reduced or oxidized (or intermediate) state [7,8], and also on the role of the possible chemical processes, which could have been induced by various sources of energy, such as intense lightning, energetic solar photons, radioactivity, intense volcanic activity, and shock waves of different kinds [7–9]. This is the so-called endogenous synthesis vision, as the prebiotic species would have been synthesized directly on Earth from simple parent molecules. This vision gained support more than 50 years ago by the ground-breaking experiment of Stanley Miller [5], an experiment having also the merit of demonstrating that an abiotic synthesis of prebiotic molecules is possible starting from very simple gaseous parent molecules [7]. For completeness, it should be mentioned that there is an alternative endogenous synthesis theory, where gas-phase reactions are not involved, according to which some organic synthesis could have taken place in the proximity of oceanic hydrothermal vents because of the relative abundance of methane and ammonia [10].

The alternative scenario is the exogenous synthesis, according to which most of the organic molecules came from space, the carriers being comets, asteroids, meteorites or even interplanetary dust particles ([11–16] and references therein). The rationale behind this suggestion is that plenty of simple organic molecules have been identified in all these objects. The idea is that they were first synthesized in the nebula from which the formation of the solar system originated. Those molecules were then incorporated in comets and meteorites far from the central star and then reached our planet [12], where they could evolve in an aqueous medium after the terrestrial temperature decreased. In favor of this scenario there is the observation of many organic molecules in the interstellar clouds, including several star-forming regions. Amongst the more than 150 molecules identified in the interstellar medium [17], in fact, only 36 do not contain carbon atoms and most of the molecules listed in Table 1 (e.g. glycolaldehyde, ethylene glycol, HCN, HC_3_N, formamide, methanimine) have been identified in the interstellar medium. In addition, complex, organic to some extent, molecules have been detected in comets, interplanetary dust particles and, especially, carbonaceous meteorites [11–16]. Following the observation, it remains to be demonstrated where and how these molecules are formed, whether they can be preserved during the early phases of solar-type star formation and in the solar nebula [11,12,18], how they can be incorporated into comets and meteorites and whether they can survive their entry into the terrestrial atmosphere [11,12]. The attractiveness of this theory is that, if it is proved that there is a link between prebiotic molecules synthesized in space and the appearance of life on Earth, a hint on the possibility that life is a widespread phenomenon in the Universe, rather than a local, fortuitous case, can be gained.

Whether in a planetary atmosphere or in the interstellar clouds (ISCs) that precede the formation of solar systems, the observation of prebiotic molecules in a wide variety of gaseous extraterrestrial objects, where physical conditions vary to a large extent, implies that gas-phase chemistry plays a role in their synthesis. Different types of molecular processes are believed to be involved, including radiative association and recombination, surface-induced processes [19], photon- or particle-induced ionization and ion-molecule reactions [20], photon- or particle-induced dissociation and radicalmolecule reactions [20]. The concomitance of all these phenomena and the complexity of their environments require a modelling approach, where all the relevant molecular processes compatible with the boundary conditions should be considered with the appropriate parameters that describe them. This strategy has been pursued by several research groups to model the chemical evolution of interstellar clouds, planetary atmospheres and cometary comae (see for instance [21–30]). For the modeling of these complex networks of elementary reactions, a number of experimental parameters are needed and, therefore, the molecular processes necessary to construct a realistic model should be fully characterized in laboratory experiments. A survey of the available literature reveals, however, that much information is still lacking if it is true that only a small percentage of the elementary reactions included in the models have been characterized in laboratory experiments under conditions that simulate those prevailing in the various environments. To understand the reason for that, let us briefly examine two typical environments where this chemistry takes place, that is, ISCs and the atmospheres of planets and satellites of our solar system.

The ISCs are essentially formed by gaseous matter [21–26,31,32], with a small percentage (1% by mass) of dust particles. The average kinetic energy is typically confined to 0.8 kJ mol^−1^ (corresponding to a temperature of 100 K) in diffuse clouds and 0.08 kJ mol^−1^ (corresponding to a temperature of 10 K) in dark molecular clouds and, therefore, the formation of molecular species via gas-phase reactions can only involve reactions without appreciable activation energy. For this reason, since the first observation of molecules in ISCs, ion–molecule reactions have been considered to play a central role [29,32], as they are usually characterized by null activation energy and can, therefore, be fast at very low temperatures. Nevertheless, only a small percentage of atomic and molecular species are ionized in the clouds. Neutral-neutral gas-phase reactions have been correctly included in the modeling of ISC chemistry only after it has been possible to measure their rate constant down to very low temperatures (as low as 15 K) by means of the CRESU (the French acronym for *Cinétique de Réaction en Ecoulement Supersonique Uniforme*) technique [33]. From these studies experimental evidence has been obtained that some reactions involving atomic or radical species (such as atomic carbon or CN, C_2_H and C_4_H radicals) are very fast, with rate constants in the gas kinetics range, even at very low temperatures [33]. Also, the results from this technique have pointed out that the extrapolation at very low temperatures of the Arrhenius dependence of the rate constant outside the range of T investigated is not warranted and important deviations from it have been observed [34,35]. The inclusion of several fast neutral-neutral reactions in the complex reaction networks that model the chemical evolution of ISCs is a recent advance in the field [22,25]. In principle, however, all the possible reactions should be investigated at the low temperature of the various interstellar objects.

After numerous missions, airborne and ground-based observations, plenty of information is available on the structure and chemical composition of the atmospheres of the planets of our solar system. Similarly to the atmosphere of Earth, the atmospheres of the other planets (or satellites, such as Titan) can be described as giant photoreactors, where the energy deposited mainly by solar photons, but also by cosmic rays and other energetic particles, drives a complex gas-phase chemistry [27,36–48]. In this case as well, to account for the chemical composition of the atmospheres, complex models including physical parameters, such as vertical transport, wind transport and temperature profiles, have been developed. These models are usually referred to as *photochemical* models, because the main source of energy that drives the various phenomena considered are solar photons. Among others, the atmosphere of Saturn’s massive moon Titan has received considerable attention because it is considered to be somewhat reminiscent of the primeval atmosphere of Earth [49,50]. In an atmosphere mainly composed by molecular nitrogen with a small percentage of methane, the detection of gas-phase nitriles (in trace amounts of a few parts per billion) has attracted a lot of interest, since these species are thought to be key intermediates towards the formation of biologically relevant molecules [1,251,^52^]. The surface temperature of Titan is ~94 K and even in the upper stratosphere the temperature does not exceed 187 K. Therefore, water is mainly in the solid state and the absence of liquid water prevents the evolution of life as we know it. On the other hand, exactly because of the absence of a biosphere, Titan provides us with the unique opportunity to investigate a chemical environment probably close enough to the primitive terrestrial atmosphere. In other words the study of the chemical evolution of Titan's atmosphere can help us to understand how biologically active molecules and their nitrile precursors can be synthesized in a mildly reducing atmosphere [49], possibly resembling that of our planet before the emergence of life drastically changed its composition. Numerous photochemical models of increasing complexity have attempted to describe the atmospheric composition of Titan and important improvements have been achieved after the results of the Cassini-Huygens mission [41–48]. Nevertheless, even the latest models [45,48] include several elementary reactions (unimolecular, bimolecular and termolecular) never studied in the laboratory, with parameters estimated by analogy with ‘similar’ ones, whereas other elementary reactions have been included with the laboratory parameters measured under different conditions (particularly temperature). The extrapolation of the experimental data to temperatures different from those actually used in the experiments is not warranted [35], while the analogy with similar systems can lead to completely erroneous evaluations [34].

In the following sections, an overview will be given of the two experimental approaches that have allowed us to reach a better description of neutral-neutral gas-phase reactions of importance in the chemical modeling of ISCs and the atmosphere of Titan. This review paper is not meant to be a comprehensive survey of the very active and productive fields of prebiotic chemistry and astrochemistry, where important contributions are also made by theoretical investigations (see, for instance, [53–55]), and the focus will be only on neutral-neutral bimolecular reactions. Firstly, the principles of the CRESU technique, that has finally allowed the measurements of several reaction rate constants at appropriately low temperatures, will be illustrated. This technique, as well as other typical kinetics techniques, usually provide us with the reactant disappearance rate constants, but more rarely are able to determine the nature of the reaction products and their branching ratio. This is, however, a very important piece of information, because in the reaction networks used in the models the products of one reaction are going to be the reagents of a subsequent one. An alternative approach is necessary and the best opportunity is furnished by free-collision experiments. This is the realm of *reaction dynamics*, a discipline which provides us with the most detailed knowledge of a gas-phase reaction and aims to verify whether a specific reaction pathway and its related products are really accessible by the system. In this respect, the crossed molecular beam (CMB) technique, especially when coupled to *universal* mass spectrometric (MS) detection, has proved to be an extraordinary tool [56]. Finally, the results on several reactive systems and their connection with the understanding of gas-phase prebiotic chemistry will be illustrated.

## 2. The CRESU Technique

**Figure 1.**
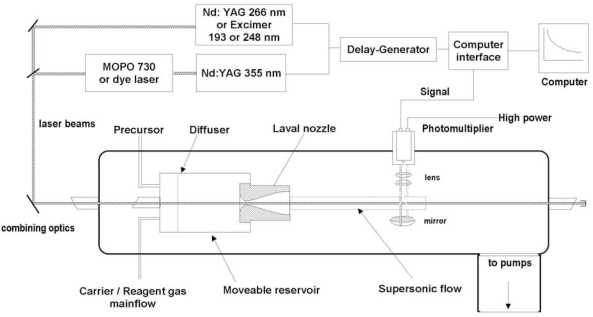
A schematic view of the PLP-LIF CRESU apparatus for the study of neutral-neutral bimolecular reaction. Reprinted from Canosa, A.; Goulay, F.; Sims, I.R.; Rowe, B.R. Gas phase reactive collisions at very low temperature: recent experimental advances and perspectives. In *Low Temperatures and cold molecules*, Copyright 2008, published with permission from World Scientific Publishing Co. Pte. Ltd, Singapore.

As pointed out in the Introduction, it is imperative to measure the rate constants of the potentially relevant reactions at the low temperatures typical of the ISCs and also of the atmospheres of the other objects of the solar system (with the only exception of Venus, all other atmospheres are characterized by an average temperature lower than that of our planet). There are two experimental approaches to cool gases to low temperatures and study their reactions: cryogenic cooling and expansion methods [33,57–59]. Cryogenic cooling is a simple approach that consists in cooling the entire reaction vessel at the desired temperature. This approach, however, is limited by the saturation vapour pressure of the reactant gases and there are practically no reactions that could be investigated at 10–20 K without condensation of the reactants on the walls of the refrigerated container. The alternative method to cool the reactant gases is the use of a supersonic expansion. The expansion from a high pressure zone to a low pressure one is an adiabatic, isentropic process which converts the thermal energy of the gas into kinetic energy. The use of a collimating axisymmetric, converging-diverging Laval nozzle, leads to the production of a cold and uniform supersonic flow of gas [33,57–59]. This is the approach used in the CRESU technique. Comprehensive descriptions of the principles of this technique can be found in several reviews [33,57–59]. An important characteristic of the expansion through a Laval nozzle is that a relatively dense medium (10^16^ – 10^17^ cm^−3^) is formed, in which the temperature and number density are constant along the axis of the flow. Different nozzles and carrier gases are used to produce a specific flow temperature and the supersonic flow regime is maintained as such also when 1–2% of additional gases are added. The chemically unstable reactants in the gas mixture (generally an atomic [60–62] or radical [63–70] species, but also unstable closed-shell species such as C_2_ [71,72]) are generated via pulsed laser photolysis (PLP) of a molecular precursor. In a typical CRESU arrangement, the decay rate of the unstable reactant is followed by laser-induced fluorescence (LIF), see Figure 1. The photolysis and probe pulsed lasers co-propagate along the axis of the supersonic flow. On the one hand, the photolysis laser produces a homogeneous concentration of the desired radical starting from a suitable molecular precursor. On the other hand, the probe laser monitors the formed radicals by exciting them to an upper electronic state. The resulting fluorescence is collected at 10–50 cm downstream of the Laval nozzle exit. For a given concentration of the reaction partner, the decay rate of the radical is observed by varying the delay time between the pulse from the photolysis laser and the pulse from the probe laser. The fluorescence signal decreases with the delay time exactly because the radicals are consumed in the reaction during their transit along the flow. A severe limit of the CRESU technique is that it cannot be used for reactions with room temperature rate constants smaller than 10^−12^ cm^3^ molec^−1^ s^−1^. This order of magnitude is imposed by the fact that the supersonic flow is maintained uniform over a distance of a few tens of centimetres. This implies a time scale of 100 – 500 μs within which the change of concentration of the atom/radical reactant has to be detected. In the classical CRESU technique, an extensive pumping system is required. To reduce the pumping system, a pulsed Laval nozzle technique was developed and used to study bimolecular reactions down to 53 K [73–83].

## 3. The Crossed Molecular Beam Technique with Mass Spectrometric Detection

Amongst the possible experimental techniques to study a bimolecular reaction under collision-free conditions, the most versatile one is the crossed molecular beam (CMB) technique with mass-spectrometric (MS) detection. This technique was first developed in the late ‘60s and used to address fundamental aspects of the reaction mechanisms at the microscopic level [56], while only recently, improvements in the production of beams of unstable species and vacuum technology have allowed the study of elementary reactions of interest in astrochemistry [31,84–135]. The main advantage of CMB experiments is that the reactants are confined into distinct supersonic beams which cross each other at a specific angle; the species of each beam are characterized by a well defined (both in magnitude and direction) velocity and are made to collide only with the molecules of the other beam, allowing us to observe the consequences of well defined molecular collisions. The products are formed at the collision center and then fly undisturbed towards the detector because of the large mean free path achieved by operating at a very low pressure (10^−5^ Pa). In CMB experiments the product detection can be done by means of spectroscopic techniques or mass-spectrometry. Even though notable examples of CMB experiments coupled to spectroscopic detection on reactions of relevance in astrochemistry are available in the literature [61,121–123,134], the use of MS detection is especially advantageous compared to spectroscopic techniques, the applicability of which requires the knowledge of the optical properties of the products – while, in many cases, their nature itself is unknown. The coupling with MS detection, in fact, makes the method *universal*, that is, applicable to the study of (at least in principle) any reaction [56]. Every species can be ionized at the electron energy typically used in the ionizer (≥70 eV) which precedes the mass filter. Therefore, it is possible to determine the mass (and, in favorable cases, the gross formula) of all possible species produced from the reactions by ionizing the product and selecting different mass-to-charge ratios (*m/z*) in the mass selector. Some problems, such as dissociative ionization and background noise, have restricted the sensitivity of the method, but the recent implementation of the *soft* electron ionization (EI) approach has partly overcome this problem [113,114,127,128]. To be noted that an important advantage with respect to common MS flow reactors is the possibility to measure product angular and velocity distributions, which allows one to directly derive the amount of the total energy available to the products and, therefore, the energetics of the reaction. This is crucial when more isomers with the same gross formula can be produced [117]. In general, the CMB technique allows one to determine (*a*) the nature of the primary reaction products, (*b*) the branching ratios of competing reaction channels, (*c*) the microscopic reaction mechanisms (*d*) the product energy partitioning. More generally, we can say that it is possible to obtain information on the underlying potential energy surfaces (PES) governing the transformation from reactants to products.

The benefits of the CMB technique strongly motivate its extension to the study of reactions of interest in astrochemistry. In the last few years the CMB method with MS detection has indeed been applied to the study of astronomically relevant reactions in different laboratories, especially in the research groups of Kaiser [31,84–112,135] and Casavecchia [113–133]. CMB machines equipped with MS detection can vary to some extent depending on the way used to generate the unstable species beams. On the one hand, we have laser-based generation techniques, amongst which laser photolysis [135] or laser induced ablation from a refractory material rod and, eventually, subsequent reaction of the ablated species with another gas [136]. This method has been widely used in the laboratory of Kaiser to produce pulsed supersonic beams of atoms or radicals, such as C and B atoms, CN and C_2_H radicals, C_2_ and C_3_ [31,87,91,94]. Alternatively, beams of unstable species can be produced by flash pyrolysis or electrical discharge. A pulsed pyrolytic source has been used by Kaiser and coworkers to produce beams of C_3_H_5_ and C_6_H_5_ radicals [137,138], while a continuous pyrolytic source has been used by Casavecchia and coworkers to produce beams of C_3_H_5_ and CH_3_ [113,139]. A radiofrequency discharge beam source has been widely used by Casavecchia and coworkers to produce beams of C, O, N, S, Cl atoms, OH and CN radicals and C_2_ [140].

**Figure 2.**
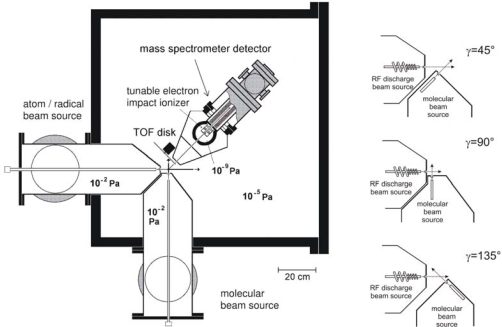
Schematic view of the Perugia crossed molecular beam apparatus (lhs). The three possible crossing beam geometries are also shown on the right-hand-side. Adapted with permission from reference [113].

An important, recent achievement in the crossed molecular beam machine with MS detection of Casavecchia is the set-up of a variable beam crossing angle configuration [113,114], see Figure 2. The relative collision energy, E_c_, in a CMB experiment is given by 1/2μv_r_^2^, where μ is the reduced mass of the system and v_r_ is the relative velocity. In general v_r_ is given by the equation v_r_^2^ = v_1_^2^ + v_2_^2^ – 2v_1_v_2_cos γ, where v_1_ and v_2_ are the two reactant beam velocities in the laboratory frame and γ is the crossing angle of the two beams. Traditionally, CMB instruments with MS detection have featured a beam crossing angle γ of 90°. In apparatuses with fixed γ = 90°, the E_c_s achievable are limited by the velocities with which the two beams can be produced. The relatively high E_c_s normally achieved in CMB experiments might affect the results obtained by this technique on astronomically relevant reactions if there is a change of the reaction mechanism with varying E_c_. To overcome this problem, a variable beam crossing angle set-up, which allows crossing of the two reactant beams at three different angles of 45°, 90°, and 135° has been implemented. With this new setting, E_c_ could be varied over a much wider range than previously possible: with the beam velocities remaining constant, the γ = 135° configuration allows reaching higher E_c_ while the γ=45° arrangement is intended for reaching very low E_c_. In this way, the bimolecular reactions can be studied under collision free conditions at collision energies that resemble those of relevance in astrochemistry with typical environments at low T.

Another important achievement obtained in the group of Casavecchia is the implementation of soft-EI by means of a tunable ionizer [113,114]. In this way, by tuning the electron energy below the threshold for dissociative ionization of interfering species, it is possible to eliminate or reduce the background or interfering signal of different sources [125,127,128] that, in several cases, would prohibit the reactive scattering experiments. Remarkably, the use of a tunable electron impact ionizer in the CMB instrument permits measuring the EI efficiency curves of the reaction products as a function of electron energy down to their ionization thresholds and, from these, obtaining a direct estimate of the ionization energy of the product radicals [132]. In other words, it is possible to measure the unknown ionization energy of radical species by ‘synthesizing’ them in CMB reactive scattering experiments.

## 4. Key Results on the Reactions of CN Radicals

Cyanopolyynes, with the general chemical formula H-(C≡C)_n_-CN, are ubiquitous in the ISCs and displzy high fractional abundances of up to 6 × 10^−9^ [141]. The simplest one, cyanoacetylene HCCCN(X^1^∑^+^), has been identified in the dark molecular clouds TMC-1 and OCM-1 as well as the carbon star IRC + 10,216 (CW Leo), towards high intensity IR sources (IRC + 10,216) and massive O-B stars within dense molecular clouds [142–144]. More complex cyanopolyynes up to HC_11_N have been assigned in TMC-1 [145,146]. Methylcyanoacetylene, CH_3_CCCN [147], vinylcyanide, C_2_H_3_CN, [148] and the two radicals C_3_N and C_5_N [149,150] have also been identified in various objects. Early models of ISC chemistry suggested that the dissociative recombination of the HCCCNH^+^ ion was the main formation mechanism of HCCCN and its isomer HCCNC. The ion HCCCNH^+^ was assumed to be formed via ion–molecule reactions, amongst which N(^4^S) + C_3_H_3_^+^. Nevertheless, this HCCCN formation route cannot explain either the observed [HCCCN]:[HCCNC]:[HNCCC] ratio or the HCCCN isotopomers (H^13^CCCN, HC^13^CCN, HCCC^13^CN) abundance towards TMC-1. Finally, a recent theoretical investigation of the possible ion-molecule reactions leading to HCCCNH^+^ showed that they are not feasible in molecular clouds [151].

Therefore, neutral-neutral reactions, based on the generalization of the reaction scheme:

(1) $$\text{CN}({\text{X}}^{2}\Sigma^{+})+\text{H}-{\left(\text{C}\equiv \text{C}\right)}_{\text{n}}-\text{H}\to \text{H}-{\left(\text{C}\equiv \text{C}\right)}_{\text{n}}-\text{CN}+\text{H}{(}^{2}{\text{S}}_{1/2})\;\;\;\;(\text{n}=1,2,\ldots )$$

have been proposed (see [152–154] and references therein).

Cyanoacetylene has also been observed in the atmosphere of Titan, together with other CN containing molecules, such as HCN, C_2_N_2_ and CH_3_CN [155–157]. Even though they occur only in trace amounts of a few parts per billion, they are of particular importance because they are thought to be the key intermediates to the formation of biologically relevant molecules. Nitriles can be hydrolyzed and react via multistep synthesis ultimately to amino acids, thus providing one of the basic “ingredients” for life. In the upper layers of Titan’s atmosphere, the energy deposition is mainly from high-energy electrons from Saturn’s magnetosphere and short-wavelength solar ultraviolet photons (λ < 155 nm). Longer wavelength photons penetrate down to the stratosphere and photodissociate HCN to cyano radicals, CN(X^2^∑^+^). The cyano radical concentration profile overlaps with that of acetylene, so that their reaction is a plausible route of formation of cyanoacetylene.

**Figure 3.**
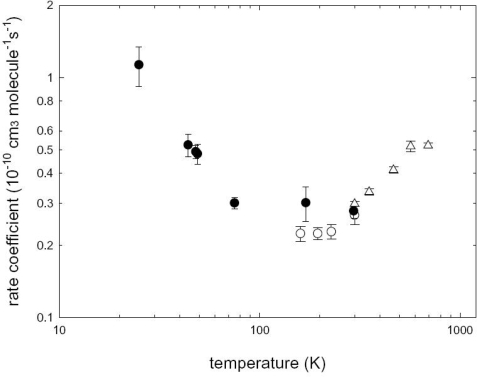
Rate coefficients for the reaction CN + C_2_H_6_ as a function of temperature, displayed on a log-log scale. The filled circles represent the results obtained in the CRESU experiment of Reference [67], open triangles are from Reference [163] while the open circles are from cooled cell experiments of Reference [67]. Adapted from Canosa, A.; Goulay, F.; Sims, I.R.; Rowe, B.R. Gas phase reactive collisions at very low temperature: recent experimental advances and perspectives. In *Low Temperatures and cold molecules*, Copyright 2008, published with permission from World Scientific Publishing Co. Pte. Ltd, Singapore.

Certainly the possible importance of CN radical reactions with the hydrocarbons widely spread in ISCs and Titan’s atmosphere became obvious after the CRESU results on these reactive systems. All the reactions with the abundant unsaturated hydrocarbons acetylene, methylacetylene, allene and ethylene [67,158] have been found to be very fast, within the gas kinetic limit, down to very low temperatures (as low as 25 K). More surprisingly, the reaction CN + C_2_H_6_, the rate constant of which decreases with decreasing temperature up to ~ 100 K, was found to become faster at temperatures below 75 K and the rate constant reaches the value of 1.1 × 10^−10^ cm^3^ molec^−1^ s^−1^ at 25 K. The temperature dependence of the reaction CN + C_2_H_6_, see Figure 3, is probably the best example to demonstrate how the extrapolation at low temperatures of the rate constant outside the range of temperature investigated can be misleading. Recent theoretical calculations have been able to account for such a peculiar behavior [159]. After the CRESU experiments provided evidence that cyano radicals easily react also at the temperatures of Titan and ISM with relatively abundant hydrocarbons, only one parameter remained to be checked: the nature and the yield of the reaction products.

By combining CMB-MS experiments with the electronic structure calculations of the relevant stationary points along the minimum energy path, Kaiser and coworkers [84–87,98] have been able to demonstrate that HCCCN + H is the only open channel for the reaction CN + C_2_H_2_ (2), thus providing an experimental confirmation of the reaction scheme (1). Also, with the same technique the reactions CN + C_2_H_4_ (3) [97], CN + CH_3_CCH (4) [96,101], CN + CH_2_=C=CH_2_ (5) [96], CN + CH_3_CCCH_3_ (6) [100], CN + C_6_H_6_ (7) [99] and CN + CH_3_CH=CH_2_ (8) [95] have been investigated. All of them share a similar reaction mechanism: the electrophilic CN radical interacts with the π electrons of the multiple bond of the unsaturated hydrocarbon without an entrance barrier, leading to the formation of a relatively stable addition intermediate. Because of its high energy content, the addition intermediate can directly undergo a C-H bond cleavage or isomerize to other intermediates before fragmenting into products. In this way, the identified molecular products were: cyanoacetylene, HCCCN, from the reaction CN + C_2_H_2_, vinylcyanide from the reaction CN + C_2_H_4_, cyanomethylacetylene, CH_3_CCCN, and cyanoallene, CH_2_CCH(CN), from the reaction CN + CH_3_CCH, cyanoallene and 3-cyanomethylacetylene from the reaction CN + CH_2_=C=CH_2_, CH_2_CCCN(CH_3_) and CH_3_CCCN from the reaction CN + CH_3_CCCH_3_, cyanobenzene from the reaction CN + C_6_H_6_ and CH_2_=CHCH_2_CN and CH_3_CH=CHCN from the reaction CN + CH_3_CH=CH_2_. The results obtained for reaction (2) have been confirmed by another CMB-MS study [117].

For the cases (2) and (3) the dominance of the H-displacement channel, leading to the molecular products listed above, has been confirmed by recent kinetic measurements where the H growth was monitored [160,161], while for the reaction of CN with propene (8) other reaction channels are open [161]. Finally, very recent experimental results obtained by the novel multiplexed time-resolved mass spectrometry with tunable synchrotron photoionization technique [162] have confirmed that C_2_H_3_CN is the sole molecular product from reaction (3), while in the case of reaction (8) the channel leading to C_2_H_3_CN + CH_3_ is the dominant one (75 ± 15%), with the minor H-displacement channel producing the isomers 1-cyanopropene (57 ± 15%), 2-cyanopropene (43 ± 15%) and 3-cyanopropene (< 15%) [162]. In all reactions discussed above, the formation of isonitriles is never favored because all the reaction channels leading to these isomers are either endothermic by a few kJ mol^−1^ or the relative exit barriers are 5 – 30 kJmol^−1^ above the energy of the separated reactants [84]. In some cases, the theoretical calculations show that the formation of HCN and an alkyl radical is a possible reaction channel, but HCN has never been observed, not even in the case of reaction (8) [162].

**Figure 4.**
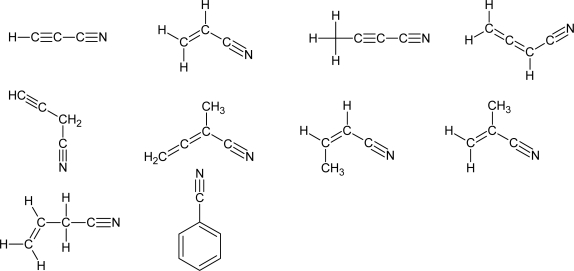
Closed-shell unsaturated nitriles observed as molecular products in the reactions between cyano radicals and unsaturated hydrocarbons.

In summary, in all the reactions investigated the CN/H exchange channel is a main reaction pathway and products with a multiple C-C bond and a –CN group are formed. The structures of the products are reported in Figure 4. Furthermore, these reactions are also very fast at very low temperatures and therefore they represent a viable route for the formation of complex nitriles in the ISCs and cold planetary atmospheres, provided that the precursor molecules (HCN and unsaturated hydrocarbons) are present. In this respect, a nice example of how the results of CMB experiments can assist the astronomical search for hitherto unobserved molecules is furnished by the detection of cyanoallene towards the dark Taurus molecular cloud (TMC-1) [164,165]. Since both cyanomethylacetylene and cyanoallene are produced from the reaction between cyano radical and methylacetylene, if the reaction (4) is the source of methylacetylene in ISCs it is reasonable to expect the presence of cyanoallene as well. And this is in fact the case [164,165].

There is some interest also in the reaction of CN with saturated hydrocarbons. In particular, the reaction with methane, the most abundant hydrocarbon in the atmosphere of Titan, CN + CH_4_ (9), was invoked to account for the formation of acetonitrile in the atmosphere of Titan [43], according to the scheme CN + CH_4_ → CH_3_CN + H. This reaction is not as fast as those with unsaturated hydrocarbons and is characterized by a room temperature rate constant of 5.95 × 10^−13^ molec cm^−3^ s^−1^ [67]. An attempt to measure the H product yield was pursued by Gannon *et al.* [161], who were not able to observe any growth in H atom production matching the kinetics of CN removal. The main reaction channel is therefore the one leading to HCN + CH_3_. An upper limit of 5% of yield for the channel leading to acetonitrile + H was given, but it was attributed to the background noise. Recent experiments by using the crossed-beam dc slice imaging [134] have been able to characterize the reaction mechanism of several reactions involving CN radicals and alkanes leading to alkyl radical + HCN.

## 5. Key Results on the Reactions of C_2_

C_2_ is one of the simplest diatomic molecules, but, in contrast to the similar N_2_ or O_2_ molecules, it is highly reactive. Another peculiarity of C_2_ is that the first electronically excited metastable state (*a*^3^Π_u_) lies only 610 cm^−1^ (or 7.3 kJ mol^−1^) above the ground state (*X*^1^∑^+^_g_). C_2_ has been identified in the interstellar medium (see for instance [166]) and cometary comae (see for instance [167–169]), while its detection in planetary atmospheres has never been reported in the literature. Nevertheless, formation and destruction processes of C_2_ are included in the photochemical models of planetary atmospheres where C_2_H_2_ has been observed [27,37–45,47,48], since C_2_ is expected to be produced (directly or in two steps) by acetylene photodissociation. Because of the very low energy content of the a^3^Π_u_ excited state, which can be produced by C_2_H photodissociation [170], the presence of C_2_(*a*^3^Π_u_) in various environments cannot be ruled out and its chemistry should be considered as well. Several experimental studies have been carried out to measure the rate coefficients of the removal of C_2_(a^3^Π_u_) and C_2_(*X*^1^∑^+^_g_) by hydrocarbons at room or higher temperature [171–174] and the results obtained for the reactions C_2_(*X*^1^∑^+^_g_) + H_2_ and C_2_(*X*^1^∑^+^_g_) + CH_4_ have been used in several photochemical models, after the extrapolation of the given rate coefficient temperature dependences to the conditions pertinent to planetary atmospheres. As already pointed out, such an extrapolation is not warranted. In the most recent model on the atmosphere of Titan by Lavvas *et al.* [47,48] the reactions of C_2_(*X*^1^∑^+^_g_) with several hydrocarbons (including acetylene) have been included with the rate constants measured at 300 K. Recent CRESU kinetic experiments in the temperature range between 24 and 300 K [71,72] on several reactions of ^1^C_2_ have now furnished the correct temperature dependence for the reactions C_2_(*X*^1^∑^+^_g_) + H_2_ and C_2_(*X*^1^∑^+^_g_) + CH_4_, and also for other reactions with simple hydrocarbons. Remarkably, those studies have demonstrated that the reactivity of C_2_(*X*^1^∑^+^_g_) with simple saturated (C_2_H_6_ and C_3_H_8_) and unsaturated (C_2_H_2_ and C_2_H_4_) hydrocarbons is very high with rate constants in the gas kinetics range. According to these new experimental results it has been established that [71]: (*a*) the reaction C_2_(*X*^1^∑^+^_g_) + CH_4_ is more efficient at low temperatures than what could be estimated by extrapolating the high temperature rate coefficients of Pitts *et al.* [173] (*2*) since the rate constants for the reactions C_2_(*X*^1^∑^+^_g_) + C_2_H_2_ and C_2_(*X*^1^∑^+^_g_) + C_2_H_4_ are larger than estimated, they should be included in the photochemical models of Titan, whereas the reaction of C_2_(*X*^1^∑^+^_g_) with H_2_ (presently included) actually has a negligible impact; (*3*) in the atmospheres of Saturn and Uranus, the reactions C_2_(*X*^1^∑^+^_g_) + C_2_H_2_ and C_2_(*X*^1^∑^+^_g_) + C_2_H_6_ should be included in the models. More recent results on C_2_(*X*^1^∑^+^_g_) + C_2_H_2_ obtained by Daugey *et al.* [175] using another CRESU apparatus at temperatures as low as 77 K confirm the results by Canosa *et al.* [71]. Also the kinetics involving C_2_ in the triplet *a*^3^Π_u_ state have been characterized with the CRESU technique [72]. Even though the trend with the temperature was quite different with respect to that of the singlet reaction, the rate constants for the reactions of C_2_(*a*^3^Π_u_) with C_2_H_2_ and other unsaturated hydrocarbons are relatively large. In conclusion, the CRESU experiments point to an efficient removal of both C_2_(*X*^1^∑^+^_g_) and C_2_(*a*^3^Π_u_) by acetylene (and other hydrocarbons) at the temperatures of relevance to Titan, Giant Planets and ISCs.

As far as the nature of the reaction products is concerned, a systematic investigation by the CMB-MS technique has been pursued by Kaiser and co-workers [88–94] for these reactions as well and quite interesting results have been obtained. For instance, it has been demonstrated that the products of the C_2_ + C_2_H_4_ reaction are not C_2_H + C_2_H_3_ or C_2_H_2_ + C_2_H_2_, as previously assumed, but *n*-C_4_H_3_ + H [92], that is, the C_2_ + C_2_H_4_ reaction leads to a carbon chain elongation. Similarly, C_4_H has been identified as the primary product of the C_2_ + C_2_H_2_ reaction, leading again to a carbon chain elongation [89,90]. Also, the 2,4-pentadiynyl-1 radical, HCCCCCH_2_, is the main product of the reaction C_2_ + CH_3_CCH [93], while the 1,3,5-hexatriynyl radical, C_6_H, is the main product of the reaction C_2_ + C_4_H_2_ (diacetylene) [88]. In all cases a carbon chain elongation was observed.

The reaction C_2_ + C_2_H_2_ has also been investigated by Casavecchia and co-workers [125,126]. In this more recent study, the C_2_ internal state population has been characterized by laser induced fluorescence to assist the interpretation of the reactive scattering results [125]. In spite of some differences in the characterization of the reaction mechanism, the results by Casavecchia and coworkers confirmed that C_4_H + H is the main reaction channel [125,126] and that C_2_ reactions with unsaturated hydrocarbons can be a viable route to synthesize polyynes in the interstellar medium.

## 6. Key Results on the Reactions of Atomic Nitrogen, N(^2^D)

The atmosphere of Titan is a gaseous environment dominated by molecular nitrogen and methane. In this condition, it is intuitive that the formation of nitriles is initiated by the reactions of active forms of nitrogen - such as nitrogen atoms or ions, which can be formed in the upper atmosphere from the abundant parent molecule N_2_. In particular, atomic nitrogen can be produced by N_2_ dissociation induced by electron impact, extreme ultra-violet photolysis and dissociative photoionization, galactic cosmic ray absorption, and N_2_^+^ dissociative recombination. These processes lead to atomic nitrogen in the ground, ^4^S_3/2_, and first electronically excited, ^2^D_3/2,5/2_, states with comparable yields. The radiative lifetimes of the metastable states ^2^D_3/2_ and ^2^D_5/2_ are quite long (12.3 and 48 hours, respectively), because the transition from a doublet to a quartet state is strongly forbidden. In addition, collisional deactivation of N(^2^D) by N_2_ is a slow process [176] and, therefore, the main fate of N(^2^D) above 800 km is chemical reaction with other constituents of Titan’s atmosphere. The production of N atoms in the ^2^D state is an important fact, because N(^4^S) atoms exhibit very low reactivity with closed-shell molecules and the probability of collision with an open-shell radical is small. On the contrary the reactions of N(^2^D) with several molecules identified in the atmosphere of Titan (including the relatively abundant CH_4_ and H_2_) can make an important contribution to the chemical evolution of the atmosphere. Yung *et al.* [41] suggested that the main products of the N(^2^D) + CH_4_ (10) reaction are NH and CH_3_ and, on this assumption, drew some reaction cycles, which ultimately lead to the formation of HCN. Also, Yung [177] suggested that the reaction N(^2^D) + C_2_H_2_ (11) can be responsible for cyanogen, C_2_N_2_, and C_4_N_2_ formation in the case it produces HCCN + H. More recent photochemical models for calculating the vertical distribution of Titan’s neutral atmosphere compounds rely on a similar suggestion [45,48].

**Figure 5.**
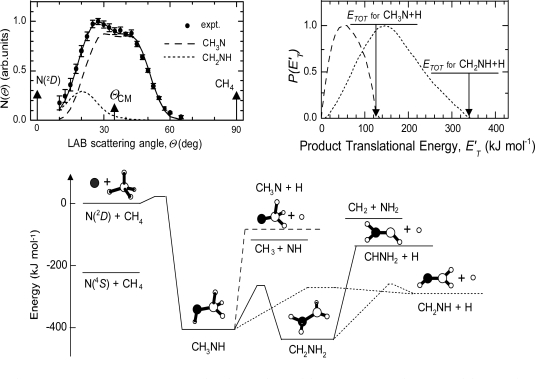
Top, left: Laboratory angular distribution of the CH_3_N/CH_2_NH products from the reaction N(^2^D) + CH_4_ at E_c_ = 37.2 kJ mol^−1^. The separate contributions to the total LAB angular distribution (solid line) from the CH_3_N channel (dashed line) and the CH_2_NH channel (dotted line) are shown; top, right: best-fit CM product translational energy distributions for the methylnitrene (dashed line) and the methanimine (dotted line) forming channels. The arrows indicate the total available energy for the various isomer channels. Bottom: schematic potential energy surface for the CH_4_N system. Reprinted with permission from reference [180].

Accurate laboratory experiments on the N(^2^D) reactions have become available only in the late 1990s, because of the experimental difficulties in studying those systems. The room temperature rate constants for reactions (10) and (11) have been found to be slightly larger than those used in the models [176]. As far as reaction (10) is concerned, however, the results of recent reaction dynamics studies do not confirm the assumptions of Yung about the reaction mechanism and the nature of the products. According to the *ab initio* calculations of the NCH_4_ PES [178], the possible products are (10a) CH_3_N + H, (10b) NH + CH_3_, (10c) CHNH_2_ + H, and (10d) CH_2_NH + H. A recent spectroscopic study under collision-free conditions [179] derived an absolute yield of 0.3 ± 0.1 for NH (from channel 10b) and 0.8±0.2 for H (from channels 10a, 10c, 10d) production. The nature of the CH_3_N isomer(s) produced in conjunction with H has been established in a CMB-MS study of the N(^2^D) + CH_4_ reaction as a function of collision energy (from ~ 20 kJ mol^−1^ to ~ 60 kJ mol^−1^) [117–119,180]. Interestingly, the channels leading to CH_2_NH (methanimine) and CH_3_N (methylnitrene) were found to be both open and the relative branching ratio, σ(CH_2_NH + H) / σ(CH_3_N + H), was found to vary considerably with the amount of energy available [119]. In Figure 5 we report the results obtained at E_c_ = 37.2 kJ mol^−1^ where significant contributions to the reaction come from both channels 10a and 10d (the channel 10c is negligible). At E_c_ = 37.2 kJ mol^−1^ σ(CH_2_NH + H) / σ(CH_3_N + H) is 0.25. A large yield of CH_3_N is not surprising at such a relatively large E_c_ and corresponds to a well-known dynamical effect: the energy which is released after the insertion of N(^2^D) into the C-H bond (corresponding to the exoergicity of 427 kJ mol^−1^ plus E_c_) is initially ‘localized’ into the two new N-H and C-N bonds of the CH_3_NH intermediate. If one of the two newly formed (and very stressed) bonds breaks apart, we have the formation of either NH + CH_3_ (corresponding to the fission of the new C-N bond) or CH_3_N + H (corresponding to the fission of the new N-H bond). The fission of one of the pre-existing C-H bonds, not directly involved in the insertion process, can only occur if some energy redistribution takes place. For this to happen, the CH_3_NH should live long enough to allow for energy rearrangement. The trend of relative branching ratio with E_c_ (it varies from 1.3 at E_c_ ˜ 20 kJ mol^−1^ to 0.03 at ˜ 60 kJ mol^−1^ [119]), confirms the above explanation: the larger is the total energy available to the system, the shorter is the intermediate lifetime and smaller the chance that the energy redistribution takes place efficiently. Notably, the trend with E_c_ for the two channels (10a) and (10b) is expected to be similar, because in both cases the fission of one of the newly formed bonds is involved. Several implications for the atmospheric chemistry of Titan follow. The assumption that only NH and CH_3_ are the products of reaction (10) is wrong since that reactive channel is minor in the laboratory experiments and, by analyzing the trend of the branching ratio as a function of the available energy, we can presume that it will be even minor under the low temperature conditions of Titan's atmosphere. Therefore, the nitrogen chemistry that relies on the dominance of the (10b) channel in the photochemical models of the atmosphere of Titan should be reconsidered. Furthermore, our results suggest that the reaction (10) is an active route of formation of methanimine, CH_2_NH, a closed-shell molecule. CH_2_NH has been recently identified in the upper atmosphere of Titan [46] and in bulk experiments that mimic the atmosphere of Titan [181]. Interestingly, CH_2_NH contains a novel C-N bond, thus demonstrating that such a bond can be generated directly by a reaction involving an active form of N_2_, the main constituent of the atmosphere of Titan, and CH_4_, the second most abundant species. The presence of an unsaturated CN bond renders CH_2_NH a reactive molecule and in a reducing environment like the atmosphere of Titan, methanimine could be converted to the fully saturated analogue methylamine. Alternatively, it could photodissociate to HCN/HNC in one or two steps. Some laboratory information is available on the UV absorption cross sections of CH_2_NH [182] and on the fragmentation of internally excited methanimine [183]. It is, therefore, possible that an important route of HCN formation in the atmosphere of Titan is:

(10d) $$\text{N}{(}^{2}\text{D})+{\text{CH}}_{4}\to {\text{CH}}_{2}\text{NH}+\text{H}$$

(12) $${\text{CH}}_{2}\text{NH}+\text{hv}(\text{UV})\to \text{HCN}+{\text{H}}_{2}(\text{or}\;2\text{H})$$

The reaction N(^2^D) + C_2_H_2_ (11) was also investigated in CMB experiments with MS detection and the experimental results were consistent with both HCCN + H and cyclic-HC(N)C + H formation [115] and essentially confirm the potential role of the N(^2^D) + C_2_H_2_ reaction as the first step in the formation of C_2_N_2_ and C_2_N_4_. Probably the case which better represents the potentiality of our experimental technique is the reaction N(^2^D) + C_2_H_4_ (13) [116]. This reaction was not considered in the early models, even though ethylene is almost as abundant as acetylene, or was considered erroneously [43]. The CMB-MS results clearly indicate that also this reaction proceeds through the formation of an addition intermediate and that products of general formula C_2_H_3_N are formed through a N/H exchange channel [116]. The experimental findings gained support from the PES calculations by Takayanagi *et al.* [184] and are consistent with the formation of ketenimine and 2*H*-azirine. It is very interesting to note, however, that according to the energy release – a piece of information which can be obtained only from a reaction dynamics experiment – a large fraction of the 2*H*-azirine and ketenimine molecules are formed with enough internal energy to spontaneously tautomerize to the most stable isomer acetonitrile [116], even in a collision free environment. Therefore, reaction (*13*) can be considered an effective route which ultimately leads to the formation of CH_3_CN. Our suggestion has been accepted and included in the models recently developed by Wilson and Atreya [45] and Lavvas *et al.* [48].

Very recent experimental results have become available on the reaction of N(^2^D) with another abundant hydrocarbon in the atmosphere of Titan, that is, C_2_H_6_. According to the preliminary analysis of the experimental distributions, C_2_H_5_N isomers have been identified as a result of an H-displacement channel [185].

**Figure 6.**
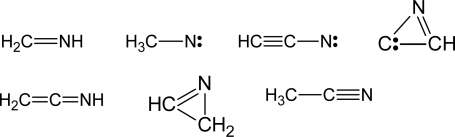
Nitriles and nitrile radicals observed as molecular products in the reactions of N(^2^D) with CH_4_, C_2_H_2_ and C_2_H_4_.

Quite interestingly, all the reactions between N(^2^D) and hydrocarbons investigated so far are characterized by the formation of active radicals (such as CH_3_N, HCCN or C_2_H_5_N), cyclic nitriles (such as 2*H*-azirine) and unsaturated nitriles (such as ketenimine, methanimine and ethanimine) (see Figure 6). These species can obviously react further with the other components of the relatively dense atmosphere of Titan. In particular, CH_2_NH can polymerize (in a similar manner as H_2_CO does) or copolymerize with the other unsaturated nitriles to form the Titan aerosol, which has been recently analyzed by the Aerosol Collector and Pyrolyser during the Huygens probe descent and found to be quite rich in nitrogen [186].

## 7. Key Results On the Reactions of Atomic Sulfur, S(^1^D)

Simple organosulfur (C-S containing) compounds have been observed in ISCs, cometary comae and also in planetary atmospheres, raising the question of how they are formed under such different conditions [187–194]. Amongst those observations, probably the most striking one is the detection of CS, CS_2_ and S_2_ after the impact of the comet Shoemaker-Levy 9 on Jupiter, where those species were observed after the impact for several days [190]. Some possible mechanisms of C-S bond formation were suggested [191] and several investigated in crossed beam experiments [192,193] and electronic structure calculations [194].

A substantial lack of experimental data, both at the level of kinetic investigation and of reaction dynamics, has so far prevented the role of the reactions of atomic sulfur with hydrocarbons or hydrocarbon radicals from being established in all of the above mentioned environments. In the light of the general interest of sulfur atom reactions and the paucity of experimental information on their reaction kinetics and especially dynamics, a systematic investigation of sulfur atom reaction kinetics and dynamics by means of the CRESU and CMB-MS techniques has been recently started [132,133]. The first results have just appeared in the literature [132,133] and revealed interesting information concerning the chemical behaviour of the sulfur atoms in the electronically excited state, ^1^D. S(^1^D) can be produced in planetary atmospheres or cometary comae by the UV photodissociation of several precursor molecules, such as OCS [198] and CS_2_ [199] and even the photodissociation of H_2_S at the Lyman-α wavelength can occur via a three body dissociation with S(^1^D) formation [200]. Also the UV photodissociation of radicals, such as SH, leads to the formation of atomic sulfur in the first electronically excited state [201]. Since the lifetime of the metastable excited state is long enough (28 s), sulfur atoms in the excited ^1^D state may well react with other gaseous species provided that the density is not too low. To be noted that the UV photodissociation of H_2_S in the primeval terrestrial atmosphere has been suggested as a possible initiating step of gas-phase prebiotic synthesis [7].

The reaction mechanisms inferred for the two reactions S(^1^D) + C_2_H_2_ and S(^1^D) + C_2_H_4_ share similar features [132,133]: the electrophilic S(^1^D) atom adds, without any barrier, to the multiple bond of acetylene or ethylene, forming an internally excited cyclic intermediate (thiirene and thiirane in the two cases). Once formed, thiirene can directly rearrange to H_2_CCS (thioketene, the most stable isomer along the singlet C_2_H_2_S PES), which, in turn, dissociates into HCCS + H. In an alternative pathway, the PES minimum configuration of thioketene is never reached and the succession of rearrangements after thiirene formation is isomerization to CC(H)SH followed by isomerization to HCCSH which, in turn, can undergo an S-H bond fission to HCCS + H. In both pathways, HCCS + H are the only possible products. Thiirane, the cyclic intermediate formed by the reaction of S(^1^D) with ethylene, can directly undergo ring-opening and three-center H_2_ elimination to thioketene (CH_2_CS) + H_2_ (the least abundant channel, with a yield of 7%) or, more readily, isomerize to vinylthiol (CH_2_CHSH) which can undergo C-H bond rupture to thiovinoxy (CH_2_CHS) + H (the second channel in importance, with a yield of 0.15). Vinylthiol can also isomerize to the slightly more stable thio-acetaldehyde (CH_3_CHS), which can undergo C-C bond cleavage to CH_3_ + HCS (the main reaction channel, with a yield of 78%). The reaction S(^1^D) + C_2_H_4_ was also investigated by the CRESU technique and was found to remain rapid down to the very low temperature of 23 K, occurring without any activation energy. This is corroborated by the theoretical calculations of the singlet C_2_H_4_S PES which did not find any appreciable reaction barrier to addition of S(^1^D) to the ethene molecule forming an initial thiirane stable intermediate. These first results on S(^1^D) reactions with hydrocarbons have shown that simple organo-sulfur compounds can be formed in relatively dense gaseous environments, provided that simple precursor molecules of sulfur atoms and UV light are available in the presence of simple hydrocarbons. These experimental findings might have several implications for the formation of prebiotic important organosulfur compounds.

## 8. Conclusions

In this paper, two powerful experimental techniques devoted to the study of bimolecular, neutral-neutral reactions have been described and their contributions to the understanding of several gas-phase reactions leading to prebiotic molecules illustrated.

It should be stressed that these experimental approaches are such that they can only focus on one elementary reaction at a time, while the complex reaction networks that describe the chemistry of interstellar clouds and planetary atmospheres comprise hundreds or thousands elementary reactions. It is, therefore, important that a collaboration is established with modelers and astronomers, so that the possibly relevant reactions and molecular species are recognized and the experimental effort is directed towards them. In this respect, positive initiatives have been two multidisciplinary EU networks, *Molecular Universe* and *Europlanet*, where physical chemists and astronomers/planetologists have had the chance to communicate and exchange ideas. Also, sensitivity methods applied to the models of ISCs and planetary atmospheres are of great help in identifying the critical reactions that should be better characterized in the laboratory to improve the reliability of the models [202–205].

## Figures and Tables

**Table 1.** Simple gas-phase molecules and their possible relation to biologically relevant molecules.

**Gas-phase molecules**	**Potential precursor of**	**Examples**
with C-N bonds	Aminoacids and Nucleobases	HCN, CH_3_CN, C_2_N_2_, HCCCN, CH_2_NH, C_2_H_3_CN
with C-O bonds	Sugars and Aminoacids	H_2_CO, CH_3_COH, (CH_2_OH)_2_
with C-C multiple bonds	Long carbon chain molecules and PAHs	From C_2_H_2_ up to polyynes
+ other molecules such as H_2_O, NH_3_, H_2_S, NH_2_CN, HCOCN, NH_2_CH_2_CN, HCONH_2_, CH_3_CONH_2_, CH_2_OHCHO, CH_3_SH etc.		
